# Methods for Designing High-Precision Relaxation Oscillator

**DOI:** 10.3390/mi16040364

**Published:** 2025-03-22

**Authors:** Zhibo Huang, Kunpeng Xu, Hongguang Dai, Zhanxia Wu, Xiaopeng Yu, Guoqiang Zhang

**Affiliations:** 1 College of Information Science & Electronic Engineering, Zhejiang University, Hangzhou 310027, China; zhibohuang2000@gmail.com; 2 College of Integrated Circuits, Zhejiang University, Hangzhou 310027, China; 13911158391@139.com (K.X.); lingshengshen@126.com (H.D.); wuzhanxia@sgchip.sgcc.com.cn (Z.W.); yuxiaopeng@zju.edu.cn (X.Y.); 3 Beijing Smartchip Microelectronics Technology Co., Ltd., Beijing 100192, China; 4 School of Information Science and Engineering, Ningbo Tech University, Ningbo 315100, China

**Keywords:** relaxation oscillator, offset voltage, delay time, temperature compensation, frequency variation

## Abstract

A novel low-power delay time cancellation (LPDTC) technique and a current ratio adjustment (CRA) method are proposed for designing high-precision relaxation oscillators. These methods effectively reduce the impacts of comparator delay time, offset voltage, and temperature-induced variations in resistors. To validate these methods, we have designed and simulated an 8 MHz open-loop relaxation oscillator using a 40 nm CMOS process. The oscillator, incorporating these advanced methods, achieves a line sensitivity of 0.38%/V and a temperature sensitivity of 43 ppm/°C over a temperature range of −40 °C to 125 °C.

## 1. Introduction

The application of microcontroller units (MCUs) has expanded significantly, now covering areas such as smart home automation, medical equipment, and motor control systems [1,2,3,4]. In this rapid development, relaxation oscillators play a crucial role, often serving as timers and primary clock sources due to their low power consumption and stable temperature performance in low-frequency applications [5]. However, the primary challenge for relaxation oscillations is minimizing the variation in comparator delay time and offset voltage caused by changes in process, supply voltage, and temperature (PVT). Many researchers have attempted to address these issues. Analog feedback loops have been used to mitigate comparator delay time variation but suffer from slow startup times and limited power efficiency [6,7,8,9]. The chopping technique can reduce the effect of offset voltage on frequency stability, but comparator delay time remains an issue [10,11]. To compensate for comparator delay, current sources with both positive and negative temperature coefficients are used [12]. Nonetheless, fluctuations in supply voltage still cause variations in comparator delay. Inverter-based oscillators provide a simple, energy-efficient solution that avoids using comparators and achieves better frequency stability, but the trigger point of the inverter is affected by PVT variation, which impacts frequency accuracy [13,14,15]. A delay-equivalent electric charge technique [16] and a small delay comparator controlled by a low-power auxiliary comparator [17] are utilized to reduce comparator delay, but they only partially address the issue. The cancellation of comparator delay successfully reduces the effects of delay and offset voltage. However, it reduces current efficiency because it requires four comparators [18].

In addition to addressing variations in comparator delay time and offset voltage, several methods have been reported to mitigate frequency errors due to temperature variations. For example, a zero-temperature coefficient current generation circuit based on a bandgap reference circuit [19], a linear temperature compensation circuit using diode-connected MOSFETs [20], a zero-temperature coefficient resistor composed of positive and negative temperature coefficient resistors [21], leakage current compensation technology [22], and digital high-order temperature compensation methods [23,24] are employed to reduce temperature-induced frequency variations. These studies highlight the importance of minimizing frequency errors with respect to temperature variations.

To address the two identified issues, we propose two methods for improving the design of high-precision relaxation oscillators in this paper:

(1) Low-power delay time cancellation (LPDTC) technique: This innovative technique minimizes the impact of the comparator delay time and offsets voltage while maintaining low current consumption by utilizing only two comparators.
(2) Current ratio adjustment (CRA) method: This method mitigates temperature-induced variations in resistors by adjusting the ratio of the reference currents in the relaxation oscillators.

## 2. Delay Time Cancellation Technique

**Figure 1:**
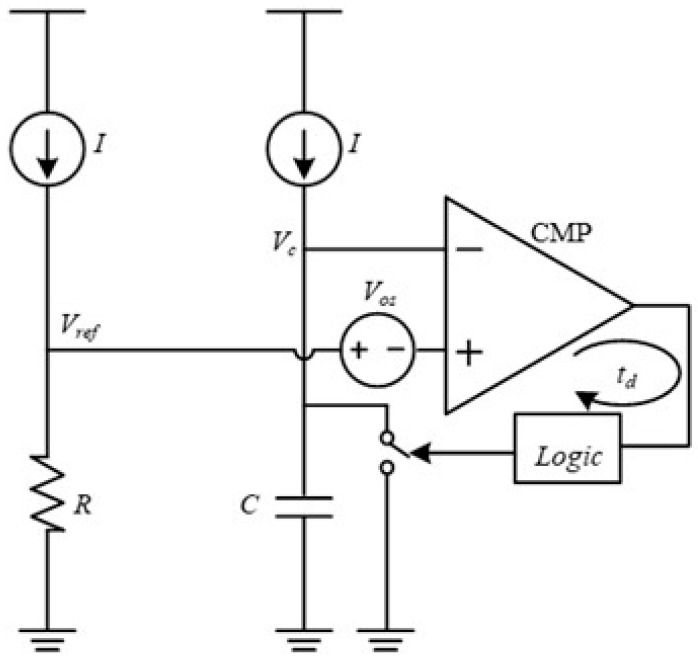
The structure of a conventional relaxation oscillator.

**Figure 2:**
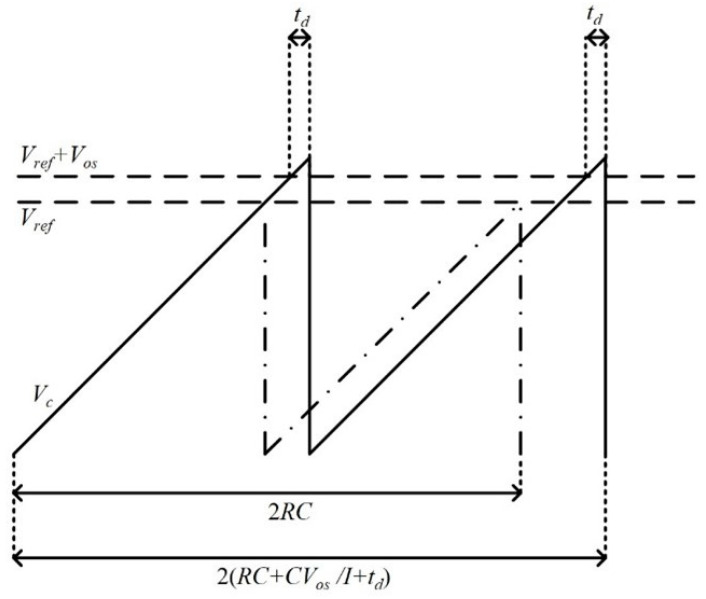
The waveform of *V_c_* in the conventional relaxation oscillator.

The basic structure of a conventional relaxation oscillator is shown in Figure 1. The period (*T_osc_*) is typically determined by the RC time constant. However, *T_osc_* is affected by the comparator offset voltage (*V_os_*) and delay time (*t_d_*). As illustrated in Figure 2, the reference voltage deviates from its original reference (*V_ref_*) due to the influence of *V_os_*. The comparator cannot react in a timely manner when the comparison voltage (*V_c_*) reaches the reference voltage (*V_ref_* + *V_os_*), resulting in a delay time *t_d_*. Hence, the equation describing the oscillator period becomes *T_osc_* = 2(*RC* + *CV_os_/I* + *t_d_*), where *I* is the bias current. *V_os_* and *t_d_* are easily affected by PVT variations, which leads to the instability of the clock period.

**Figure 3:**
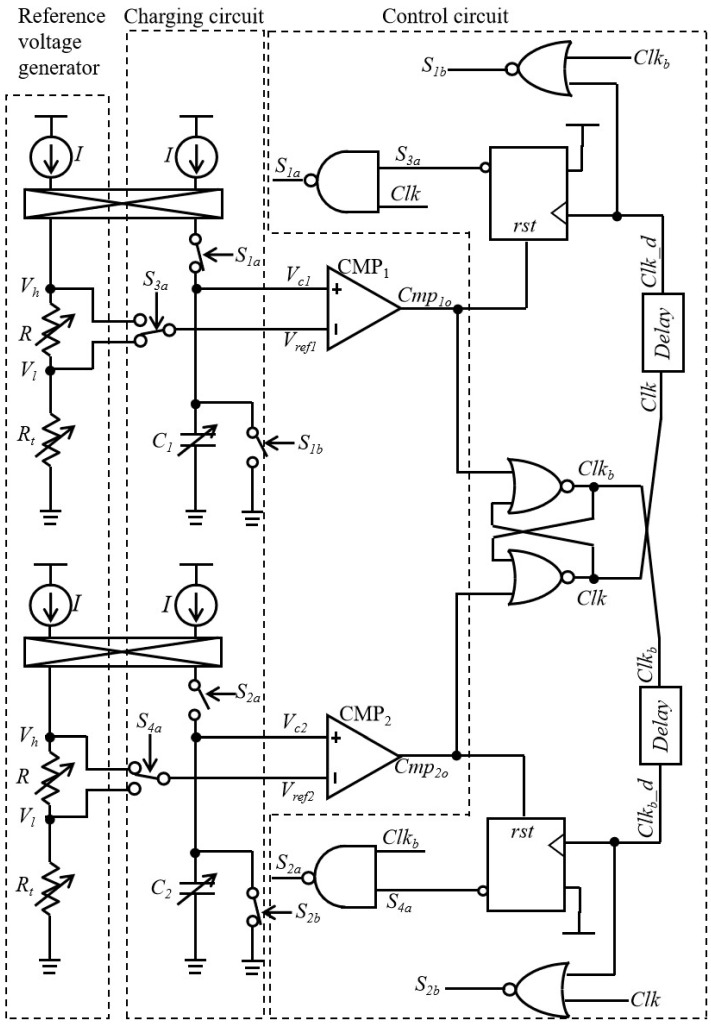
The structure of the relaxation oscillator using the LPDTC technique.

**Figure 4:**
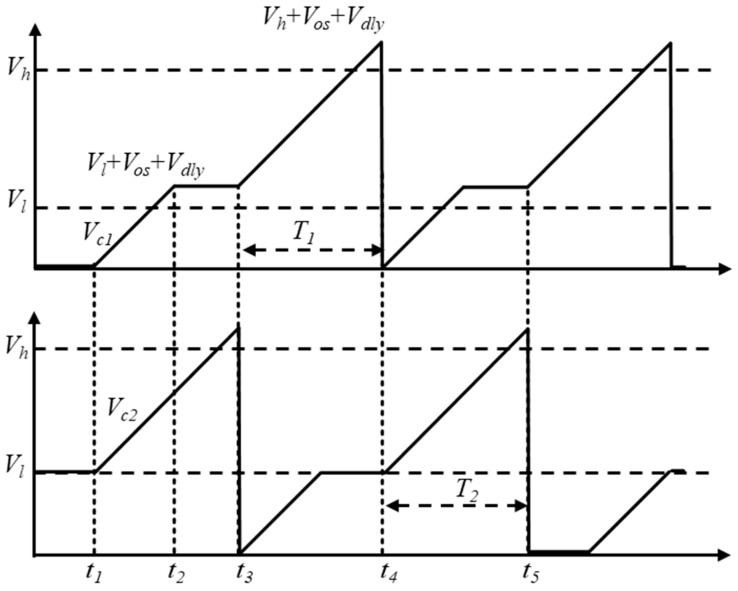
The principle of the LPDTC technique.

To eliminate the impact of comparator delay time and offset voltage, the LPDTC technique is proposed and implemented in the relaxation oscillator (Figure 3). The principle of the LPDTC technique can be illustrated in Figure 4. In the first charging phase, from *t*_1_ to *t*_2_, *V_ref_*_1_ is equal to *V_l,_* and the capacitor *C*_1_ is charged until *V_C_*_1_ reaches *V_l_* + *V_os_* + *V_dly_* by current *I* shown in Figure 3 (where *V_dly_* = *It_d_*/*C*_1_). Subsequently, before further charging to *V_h_* + *V_os_* + *V_dly_*, a brief waiting period is required from *t*_2_ to *t*_3_. Following this, the second charging phase of *C*_1_ occurs from *t*_3_ to *t*_4_, with a certain duration

(1) $$T_{1}=\frac{\left(V_{h}+V_{os}+V_{dly})-(V_{l}+V_{os}+V_{dly}\right)}{k}=\frac{\left(V_{h}-V_{l}\right)}{k}$$

where *k* represents the slew rate, equal to *C*_1_/*I*. Since *C*_2_ experiences the same charge process as *C*_1_, we can get *T*_2_ = *t*_5_ − *t*_4_ = *T*_1_ in the condition of *C*_1_ = *C*_2_ = *C*. Therefore, the clock period of the proposed oscillator is

(2) $$T_{osc}=\frac{1}{f_{0}}=2T_{1}=\frac{2C(V_{h}-V_{l})}{I}=2RC$$

As shown in Equation (2), *T_osc_* is not affected by *V_os_* and *t_d_*. Thus, the proposed LPDTC technique can improve the frequency variation of the oscillator caused by changes in *V_os_* and *t_d_*.

It is important to note that while capacitor leakage currents and charge injection effects from MOS switches, such as *S*_1*a*_ and *S*_1*b*_, can potentially influence the clock period, their impact is minimized in this study. To address these issues, we employ Metal–Organic–Metal (MOM) capacitors to significantly reduce leakage currents. Furthermore, capacitors *C*_1_ and *C*_2_ are sized approximately 100 times larger than the parasitic capacitance of the MOS switches, effectively mitigating the charge injection effects. As a result, the influence of these factors on the clock period is minimal.

**Figure 5:**
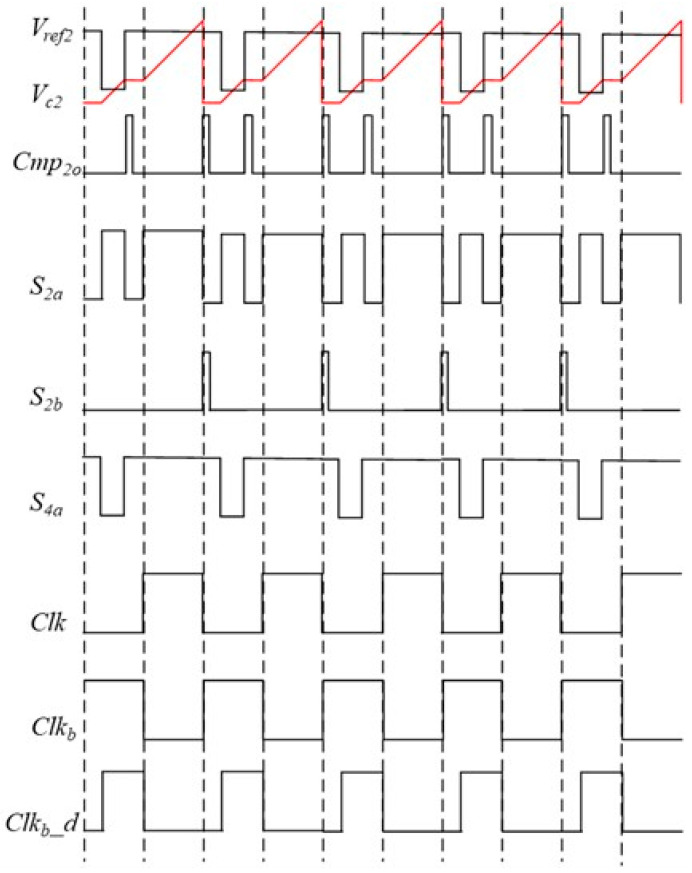
The waveforms of the proposed oscillator.

To achieve the above operation, a specialized control circuit is designed to control the operational status of the proposed oscillator shown in Figure 3. The action waveforms of this circuit are shown in Figure 5. At the beginning of one period, the signal *S*_4*a*_ is set high to make *V_ref_*_2_ equal to *V_h_*. When the rising edge of *Clk_b__d* occurs, *V_ref_*_2_ falls to *V_l_* because *S*_4*a*_ is set low. Meanwhile, *S*_2*a*_ is changed to high to start the first charging phase of the capacitor *C*_2_. In this phase, the comparator delay time is converted to voltage and stored. After *V_c_*_2_ exceeds *V_ref_*_2_, *Cmp*_2*o*_ is triggered, making *S*_2*a*_ change to low and *S*_4*a*_ to high. Under this condition, the charging of capacitor *C*_2_ stops, and *V_c_*_2_ is held. When the rising edge of *Clk* occurs, the capacitor *C*_2_ is charged again by controlling *S*_2*a*_. As described in Equation (1), the time for this charging phase is not affected by comparator delay. The action waveforms of the circuit surrounding CMP1 exhibit a 180-degree phase difference, similar to those shown in Figure 5. These periodic actions enable the clock signal to output continuously, remaining unaffected by the comparator delay time and offset voltage.

**Figure 6:**
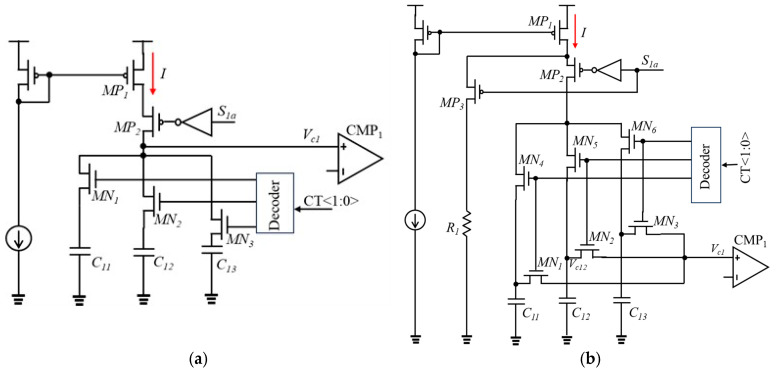
The structure of the charging circuit: (**a**) conventional structure; (**b**) proposed structure.

**Figure 7:**
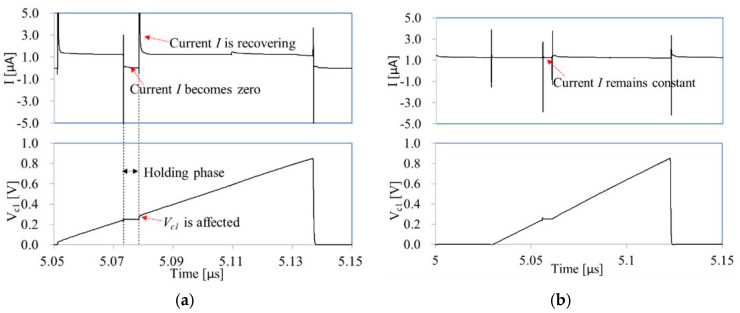
The waveforms of the charging current (**a**) in the conventional charging circuit, and (**b**) in the proposed charging circuit.

The optimization of charging circuits is necessary for the relaxation oscillator using the LPDTC technique. Figure 6a illustrates the conventional structure of a charging circuit. During the holding phase, the current *I* becomes zero (as shown in Figure 7a) because MP_1_ enters the linear region when *S*_1*a*_ is at a low level. When *S*_1*a*_ transitions back to a high level, the current *I* needs time to recover, resulting in a variation in *V_c_*_1_. Additionally, the resistance of switches MN_1_, MN_2_, and MN_3_ in the variable capacitor impacts the charging time. To address these issues, a novel charging circuit is proposed, as shown in Figure 6b. When *S*_1*a*_ is at a low level, current *I* flow to *R*_1_, ensuring that MP_1_ remains in the saturation region. Consequently, the current *I* remains constant when *S*_1*a*_ changes (as shown in Figure 7b). It should be noted that MP_2_ and MP_3_ are not controlled by a non-overlap clock due to the impact of the delay of the clock on its frequency. Furthermore, the structure of the variable capacitor is optimized. The resistance of the switches MN_1_, MN_2,_ and MN_3_ does not impact the charging time, because the currents flowing through them are zero. For instance, when switches MN_5_ and MN_2_ are turned on, capacitor *C*_12_ is selected. In this scenario, *V_c_*_1_ is equal to *V_c_*_12_ because no current flows through MN_2_. Furthermore, the resistance of switch MN_5_ also does not influence the charging time since the sampled voltage is *V_c_*_12_ rather than the voltage at the drains of the MOS transistors depicted in Figure 6a.

**Figure 8:**
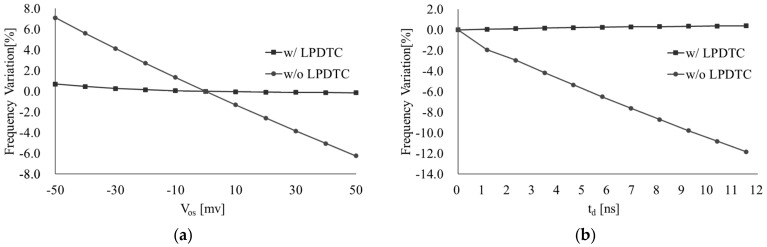
The variation in the frequency (**a**) with the change in *V_os_* and (**b**) *t_d_*.

The simulation results illustrating the effects of *V_os_* and *t_d_* are presented in Figure 8. When *V_os_* varies from −60 mV to 60 mV, the frequency variation of the oscillator using LPDTC is improved to 0.85%, compared to 13.34% without LPDTC. Similarly, with a variation in *t_d_* of 12 ns, the frequency variation in the oscillator using LPDTC is improved to 0.38%, compared to 12.1% without LPDTC. These simulation results demonstrate the effectiveness of the LPDTC technique in mitigating frequency variation caused by *V_os_* and *t_d_*.

## 3. Current Ratio Adjustment Method

**Figure 9:**
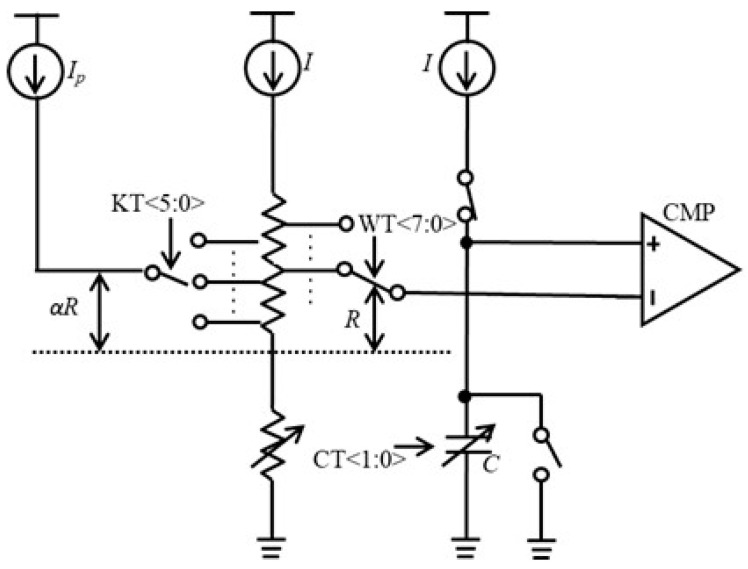
Proposed CRA circuit.

Implementing temperature compensation in the relaxation oscillator is crucial due to the temperature-induced variations in resistor *R*, which affect the frequency, as shown in Equation (2). In this work, we propose the current ratio adjustment (CRA) method to compensate for these variations by adjusting the current ratio, as illustrated in Figure 9. This approach contrasts with the temperature coefficient adjustment of reference currents employed in certain bandgap reference (BGR) circuits [25,26]. In those BGR circuits, the adjustment of the temperature coefficient of the reference current is achieved by modifying the values of resistors that determine the reference current through MOS switches. However, since these MOS switches are connected in series or parallel with the resistors, their resistance can impact the temperature coefficient of the reference currents. Consequently, the size of these MOS switches must be sufficiently large to mitigate this influence, which in turn increases the overall size of the circuit. In contrast, our proposed CRA method does not involve altering the reference current itself, providing an advantage by effectively mitigating the impact of the resistance of the MOS switches on both the reference current and the overall circuit size. The proposed circuit introduces a positive temperature coefficient of absolute temperature (PTAT) current [25,26,27,28,29], *I_p_*. The frequency of the oscillator can be expressed as

(3) $$f_{0}=\frac{1}{2RC}\cdot \frac{I}{I+\alpha I_{p}}$$

where *α* is the resistance ratio shown in Figure 9. Assuming *I* is a temperature-insensitive current and all higher-order (≥2) temperature coefficients are zero, we have

(4) $$\begin{array}{l} f_{0}=\frac{1}{2RC}\cdot \frac{I}{I+\alpha I_{p}}\\ =\frac{I}{2R_{0}C(1+\lambda_{R}\Delta T)[I+\alpha I_{p0}(1+\lambda_{Ip}\Delta T)]}\\ =\frac{I}{2R_{0}C(I+\alpha I_{p0})}\cdot \frac{I}{1+\Delta T(\lambda_{R}+\frac{\lambda_{Ip}}{I_{ratio}+1})} \end{array}$$

where *I_ratio_* is equal to *I*/*αI_p_*_0_; *R*_0_ and *I_p_*_0_ are the values of *R* and *I_p_* at room temperature, respectively; and *λR* and *λI_p_* are the first-order temperature coefficients (TC1) of *R* and *I_p_*, respectively. It can be obtained from Equation (4) that when *I_ratio_* satisfies Equation (5), *f*_0_ becomes insensitive to temperature.

(5) $$I_{ratio}=-(\frac{\lambda_{Ip}}{\lambda_{R}}+1)$$

**Figure 10:**
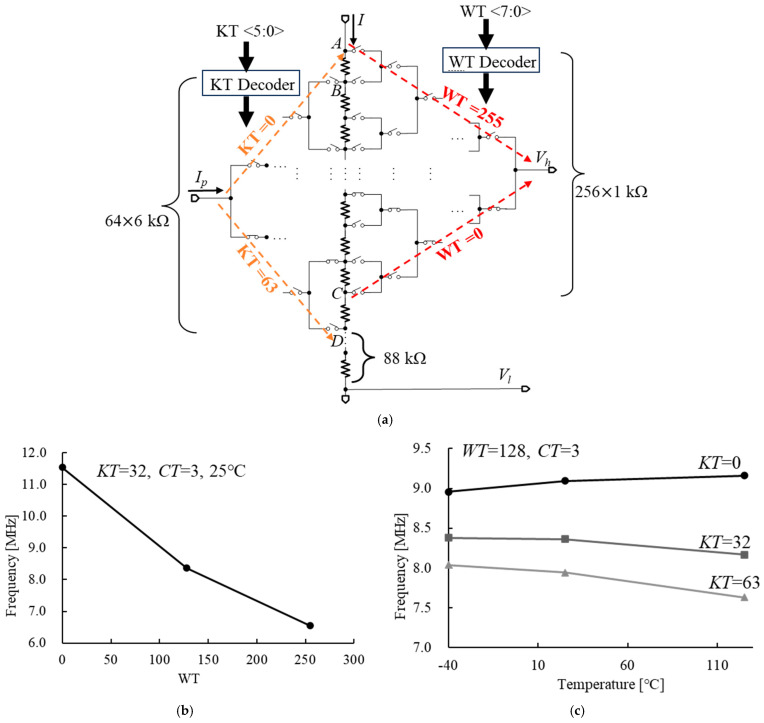
(**a**) The structure of the variable resistor *R*, (**b**) the example results of adjusting *WT*, and (**c**) the example results of adjusting *KT*.

As depicted in Figure 9, we can adjust *α* by modifying the 6-bit temperature coefficient control word *KT*, thereby adjusting *I_ratio_*. The circuit involving the variable resistor *R* is illustrated in Figure 10a. The switches are controlled by *KT* and *WT* through their respective decoders. When *KT* is set to zero, *I_p_* flows through node B. Conversely, when *KT* is set to 63, *I_p_* flows through node D. Additionally, when *WT* is set to 255, the voltage *V_h_* corresponds to the voltage at node A. In contrast, when *WT* is set to zero, *V_h_* reflects the voltage at node C. The example results obtained from varying the values of WT and KT are illustrated in Figure 10b and Figure 10c, respectively.

From Figure 10, we can calculate

(6) $$\alpha =\frac{88+6KT}{280+WT}$$

where *WT* is the 8-bit control word used to adjust the absolute frequency of the oscillator.

**Figure 11:**
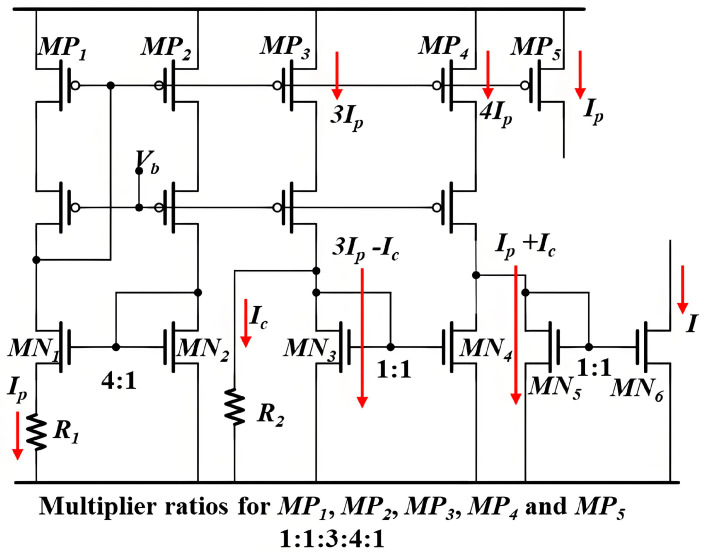
Proposed reference current generator.

The currents *I* and *I_p_* are provided by the proposed reference current generator depicted in Figure 11. Since MN_1_ and MN_2_ operate in the subthreshold region, *I_p_* can be expressed as

(7) $$I_{p}=\frac{\zeta }{R_{1}}\cdot \frac{kT}{q}\cdot \ln n$$

where *ζ*, *k*, and *q* are the subthreshold slope factor, Boltzmann constant, and elementary charge, respectively. Then, *I* can be expressed as

(8) $$I=I_{p}+I_{c}=\frac{\zeta }{R_{1}}\cdot \frac{kT}{q}\cdot \ln n+\frac{V_{GS2}}{R_{2}}$$

Given that MN_3_ also operates in the subthreshold region, *I_c_* is a complementary-to-absolute-temperature (CTAT) current. Consequently, the temperature-insensitive current *I* can be achieved.

**Figure 12:**
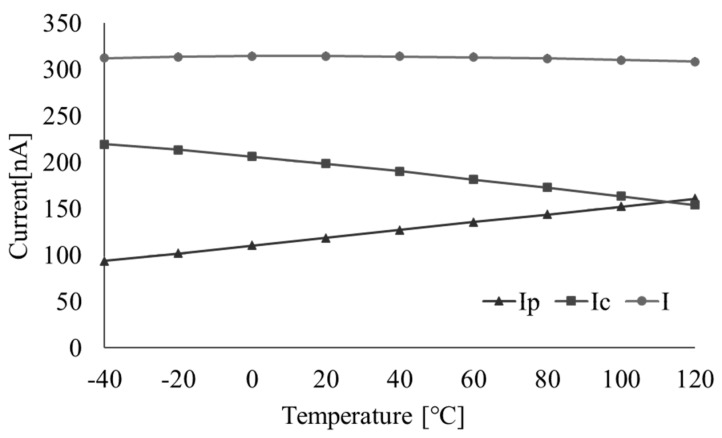
Simulation results of *I_p_*, *I_c_*, and *I*.

Figure 12 illustrates the simulation results of *I_p_*, *I_c_*, and *I* as functions of temperature, confirming that their temperature characteristics align with the theoretical predictions.

**Figure 13:**
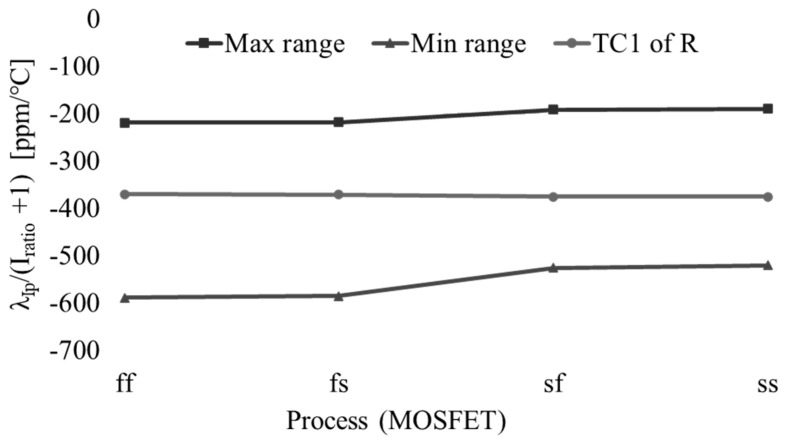
Adjustable range of *λI_p_*/(*I_ratio_* + 1).

The adjustable range of *λI_p_*/(*I_ratio_* + 1) is presented in Figure 13. This range can accommodate the TC1 of the resistor *R* with a margin exceeding ±100 ppm/°C across all MOSFET corners. This implies that a temperature-insensitive oscillation frequency can be obtained at each MOSFET corner by adjusting the control word *KT*.

**Figure 14:**
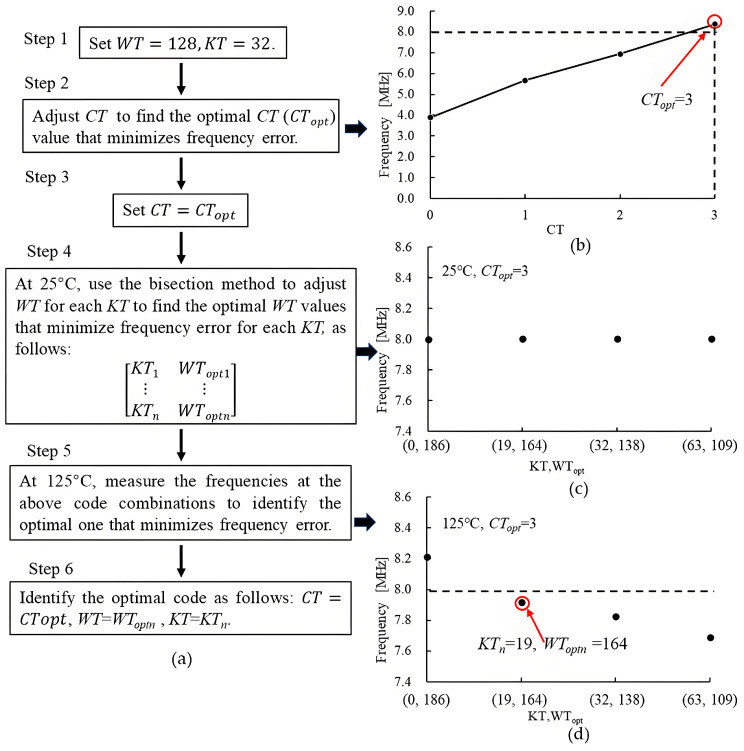
(**a**) The frequency trimming process of the proposed oscillator, and the example results of (**b**) Step 2, (**c**) Step 4, and (**d**) Step 5.

The frequency trimming process is illustrated in Figure 14a. Initially, *WT* and *KT* are set to 128 and 32, respectively. Next, *CT* (the control words of the variable capacitor shown in Figure 6a) is adjusted to find the optimal value (*CT_opt_*) that minimizes frequency error. Figure 14b presents an example of the results obtained from this step. Then, *CT* is set to *CT_opt_*, and *WT* is adjusted using the bisection method for each *KT* to find the optimal *WT* values that minimize frequency error for each *KT* at 25 °C, as follows:

(9) $$\left[\begin{array}{ccc} KT_{1} & & WT_{opt1}\\ \vdots & & \vdots \\ KT_{n} & & WT_{optn} \end{array}\right]$$

Figure 14c presents an example of the results obtained from this step.

Subsequently, at 125 °C, the frequencies for the above code combinations are measured to identify the optimal one that minimizes frequency error. Figure 14c presents an example of the results obtained from this step. Finally, the optimal code is identified as *CT* = *CT_opt_*, *WT* = *WT_optn_*, and *KT* = *KT_n_*. The trimming resolution of *WT* is approximately 0.15%, and that of *KT* is 6.25 ppm/°C. Therefore, the trimming error is about 0.13% across the temperature range from −40 °C to 125 °C.

## 4. Simulation Results

**Figure 15:**
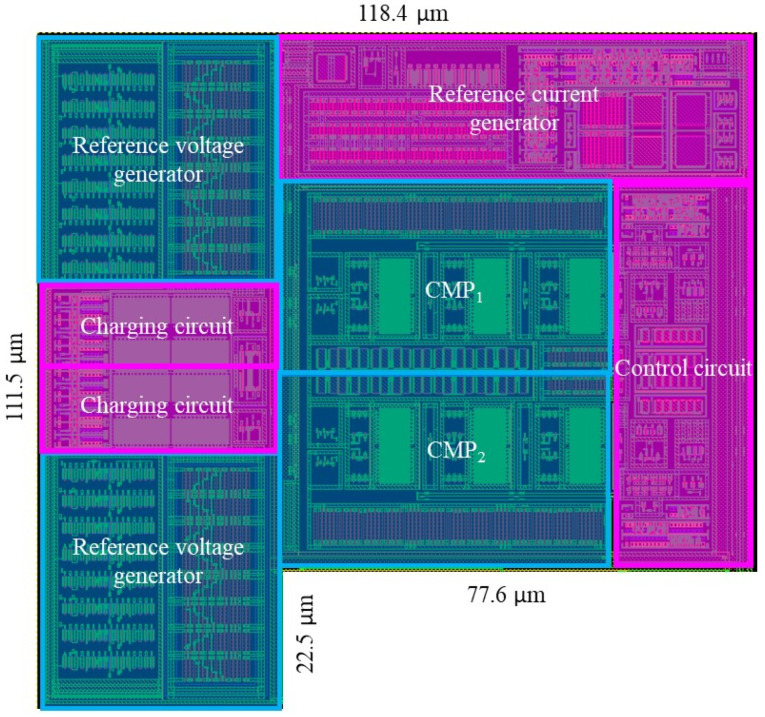
The layout of the oscillator.

The proposed LPDTC technique and CRA method are implemented in an 8 MHz relaxation oscillator designed using a 40 nm CMOS process. Figure 15 shows the layout of the proposed oscillator.

**Figure 16:**
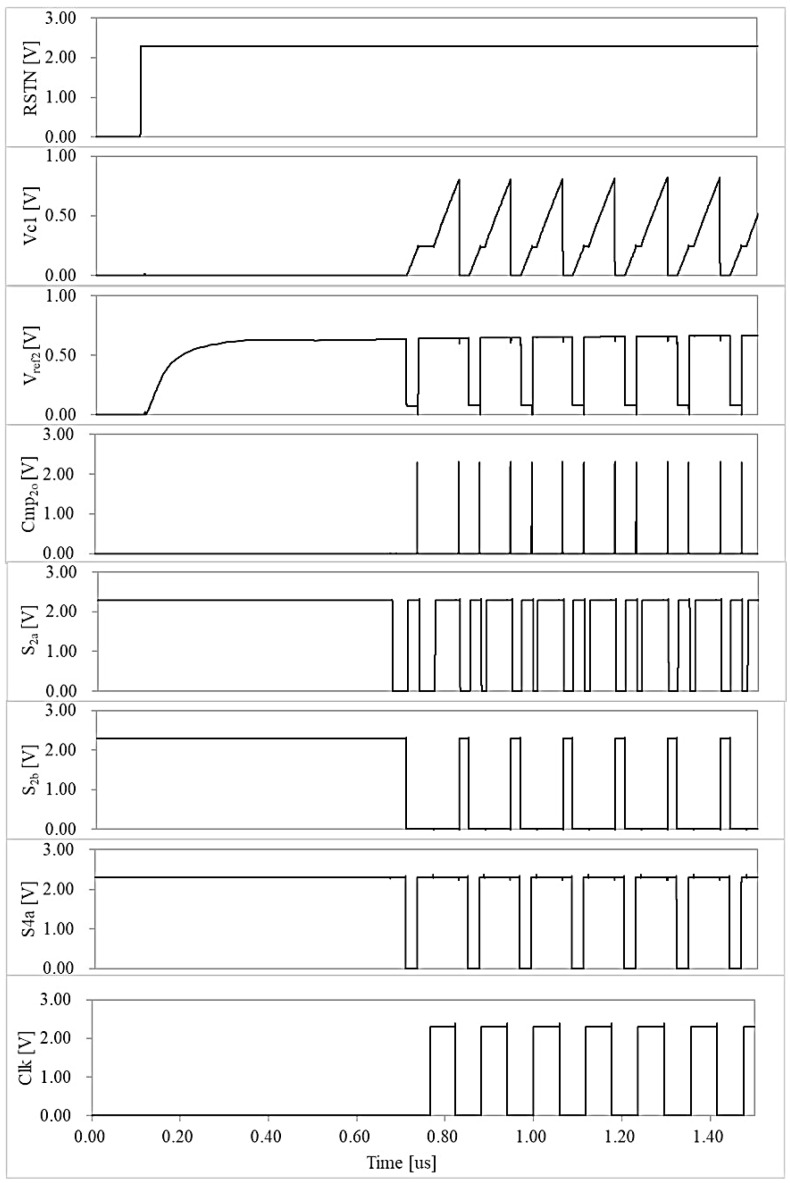
The simulated waveforms following the reset of the oscillator.

**Figure 17:**
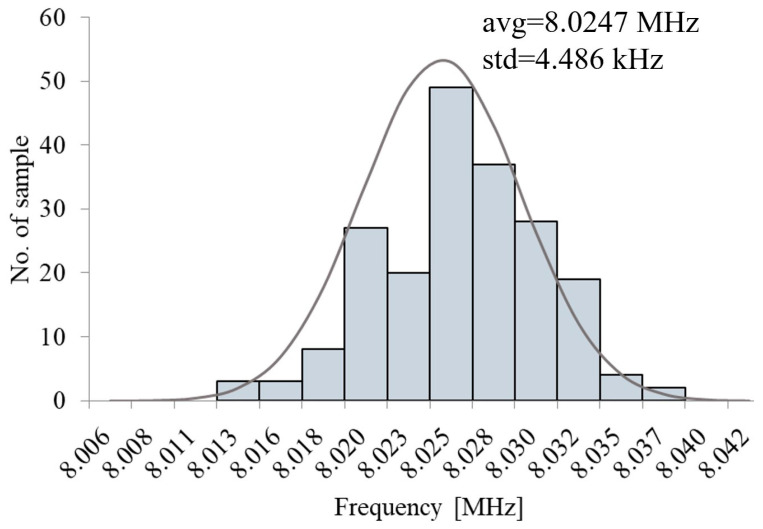
The Monte Carlo simulation result of the oscillator.

**Figure 18:**
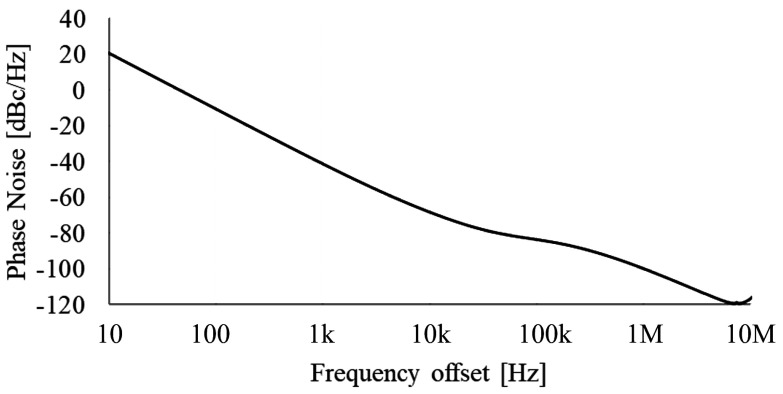
The phase noise simulation result of the oscillator.

**Figure 19:**
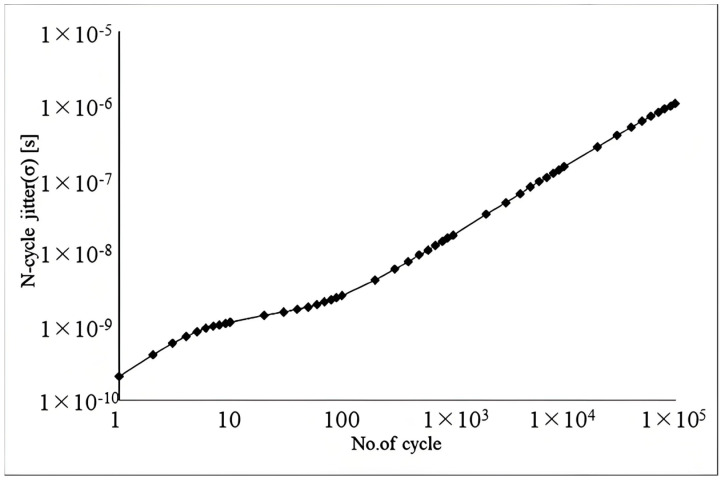
The N-cycle jitter simulation result of the oscillator.

**Figure 20:**
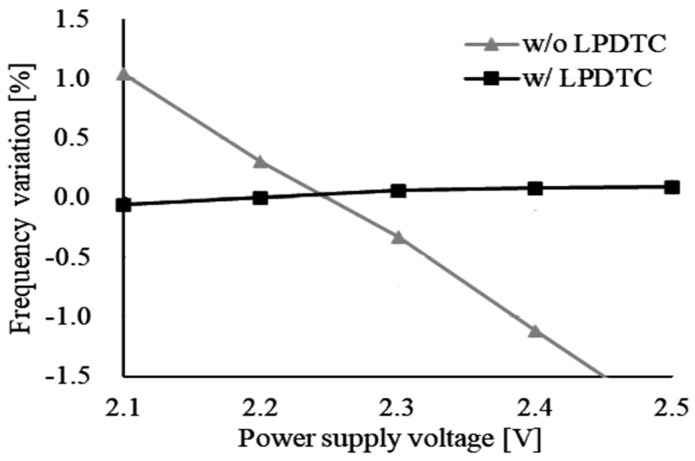
Frequency variation due to power supply fluctuations.

**Figure 21:**
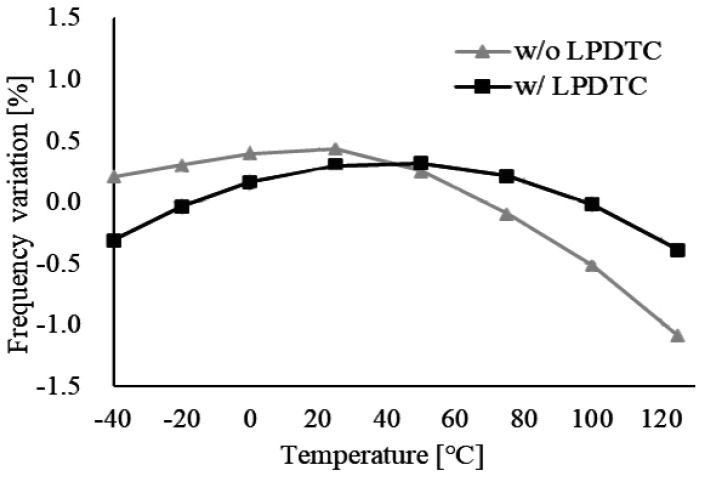
Frequency variation due to temperature fluctuations.

Figure 16 displays the waveforms following the reset of the oscillator. It is evident that by the second clock cycle post-reset, the control circuit functions as shown in Figure 5, resulting in a stabilized clock period. The frequency standard deviation is 4.486 kHz, as shown by the 200-point Monte Carlo simulation result in Figure 17. The phase noise results presented in Figure 18 have been obtained through Periodic Steady-State (PSS) and Phase Noise (Pnoise) simulations. The N-cycle jitter $\sigma_{Nj}$ illustrated in Figure 19 can be calculated by integrating the phase noise, as described in Equation (10)

(10) $$\sigma_{Nj}=\frac{\sqrt{2}}{\pi f_{0}}\cdot \sqrt{\int_{100\mathrm{Hz}}^{1\mathrm{GHz}}L(\Delta f)\cdot \sin^{2}(\pi \Delta f\cdot \frac{N}{f_{0}})}$$

where L(Δf) represents the phase noise of the oscillator at a frequency offset Δ*f*, and *N* denotes the number of cycles. Figure 20 demonstrates that with the LPDTC technique, the frequency variation due to power supply voltage fluctuations is improved to approximately 0.15%, compared to over 2% without it. Furthermore, Figure 21 illustrates that after the temperature compensation using the CRA method, the frequency variation due to temperature changes is reduced to approximately ±0.4% with the LPDTC technique, compared to ±0.8% without it. Although the second-order temperature dependence observed in Figure 21 is a result of neglecting all higher-order temperature coefficients in the CRA method, as indicated in Equation (4), the frequency variation due to temperature changes remains sufficiently small for most applications of relaxation oscillators. The performance comparison with other designs is presented in Table 1, which shows that the proposed oscillator can operate at a higher frequency with low frequency variation and low current consumption.

## 5. Conclusions

This paper presents an innovative LPDTC technique and a CRA method, which effectively mitigate comparator delay time, offset voltage and temperature-induced variations in resistors. These methods are implemented in an 8 MHz relaxation oscillator designed using a 40 nm CMOS process. As a result, the oscillator achieves a line sensitivity of 0.38%/V and a temperature sensitivity of 43 ppm/°C over a temperature range of −40 °C to 125 °C.

## Figures and Tables

**Table 1:** Performance comparison with other designs.

Reference	[11]	[12]	[14]	[15]		[16]	[17]	[18]	[22]
Process [nm]	65	180	180	55		180	500	350	65
Supply Voltage [V]	0.95~1.05	1.2~2.4	1.0~2.0	0.6~1.1		1.15~1.25	3~5.5	3~4.5	0.35~0.8
Temp. Range [°C]	−40~90	−20~80	−40~85	−40~125		−40~125	−55~125	−40~125	−40~85
Frequency [Hz]	18.5 k	1.1 M	100 k	33 k		2.9 M	1 M	1 M	4.2 k
Current Eff. [μA/MHz]	7	0.4	5.4	9.24		2.1	-	63.6	4.88
Temp. Sen. [ppm/°C]	85	64	51	58		45	124	48	114
Line Sen. [%/V]	5	3	0.4	0.75		1.245	0.16	0.28	5.45
Phase Noise @ 100 kHz [dBc/Hz]	-	−79.48	-	-		-	−115.07	-	-
Period Jitter (σ) [ps]	-	-	-	-		88.96	170	-	-
Allen Floor [ppm]	20	-	-	-		-	-	-	-
FoM_1_ [dB]	111.5	121.1	112.7	112.6		116.1	110.2	96.8	117.1
FoM_2_ [dB]	173.4	183.0	176.6	177.1		181.7	171.8	162.1	177.5
FoM_3_ [dB]	-	131.0	-	-		155.3	155.3	-	-
Area [mm^2^]	0.105	0.075	-	0.052		-	-	0.04	0.34
Result Type	Measured	Measured	Pre-sim	Post-sim		Post-sim	Measured	Measured	Measured
**Reference**	**[19]**	**[30]**	**[31]**		**[32]**		**[13]**	**[33]**	**This Work**
Process [nm]	110	130	65		28		180	130	40
Supply Voltage [V]	3.3	0.99~1.01	0.95~1.45		0.35~0.38		1.4~2	1.4~1.6	2.1~2.5
Temp. Range [°C]	−40~125	40~80	0~90		−20~120		−40~125	20~60	−40~125
Frequency [Hz]	10 M	1.2 M	3 M		2.1 M		10.5 M	3.2 M	8 M
Current Eff. [μA/MHz]	-	4.83	5.77		1.85		14.95	8.48	4.1
Temp. Sen. [ppm/°C]	133.3	296	133		158		137	1253	43
Line Sen. [%/V]	-	3.6	0.6		26.8		4.4	0.4	0.38
Phase Noise @ 100 kHz [dBc/Hz]	-	-	−114		-		−115.6	-	−83.5
Period Jitter (σ) [ps]	-	-	50		800		9.86	455	214
Allen Floor [ppm]	-	-	-		-		-	140	-
^1^ FoM_1_ [dB]	-	113.2	112.4		121.8		106.8	109.2	110.4
^1^ FoM_2_ [dB]	-	169.2	170.7		181.2		167.6	154.2	176.2
^1^ FoM_3_ [dB]	-	-	161.2		-		162.6	-	132.9
Area [mm^2^]	-	0.016	0.044		0.005		0.015	0.073	0.011
Result Type	Pre-sim	Measured	Measured		Measured		Measured	Measured	Post-sim

^1^ FoM_1_, FoM_2_, and FoM_3_ are defined as in [17].

## Data Availability

The original contributions presented in this study are included in the article. Further inquiries can be directed to the corresponding author.
